# Anatomy-Correlated Breast Imaging and Visual Grading Analysis Using Quantitative Transmission Ultrasound*™*

**DOI:** 10.1155/2016/7570406

**Published:** 2016-09-26

**Authors:** John C. Klock, Elaine Iuanow, Bilal Malik, Nancy A. Obuchowski, James Wiskin, Mark Lenox

**Affiliations:** ^1^QT Ultrasound Labs, Novato, CA 94949, USA; ^2^Department of Quantitative Health Sciences, Cleveland Clinic Foundation, Cleveland, OH 44195, USA

## Abstract

*Objectives*. This study presents correlations between cross-sectional anatomy of human female breasts and Quantitative Transmission (QT) Ultrasound, does discriminate classifier analysis to validate the speed of sound correlations, and does a visual grading analysis comparing QT Ultrasound with mammography.* Materials and Methods*. Human cadaver breasts were imaged using QT Ultrasound, sectioned, and photographed. Biopsies confirmed microanatomy and areas were correlated with QT Ultrasound images. Measurements were taken in live subjects from QT Ultrasound images and values of speed of sound for each identified anatomical structure were plotted. Finally, a visual grading analysis was performed on images to determine whether radiologists' confidence in identifying breast structures with mammography (XRM) is comparable to QT Ultrasound.* Results*. QT Ultrasound identified all major anatomical features of the breast, and speed of sound calculations showed specific values for different breast tissues. Using linear discriminant analysis overall accuracy is 91.4%. Using visual grading analysis readers scored the image quality on QT Ultrasound as better than on XRM in 69%–90% of breasts for specific tissues.* Conclusions*. QT Ultrasound provides accurate anatomic information and high tissue specificity using speed of sound information. Quantitative Transmission Ultrasound can distinguish different types of breast tissue with high resolution and accuracy.

## 1. Introduction

Breast structure is composed of thousands of terminal ductolobular units (TDLU) and their supporting connective tissue [1]. X-ray mammography (XRM) is the most common breast imaging modality, but when used routinely, it cannot define anatomic detail well enough to visualize TDLU. Although sonographic resolution using hand-held ultrasound (HHUS) can visualize several orders of mammary ducts and the functional unit of the breast, the TDLU [2], the technology is limited in the depth to which it can penetrate with high resolution and in its limitation in performing image acquisition in only 2 dimensions (2D). There is still no clinically available sonographic method to perform true 3-dimensional image acquisition and true 3-dimensional (3D) reconstruction of the breast at high resolution. We present images using Quantitative Transmission Ultrasound (QT Ultrasound) as a novel technology that performs true 3-dimensional imaging and reconstruction at high resolution.

QT Ultrasound provides high resolution breast imaging without ionizing radiation, compression, or contrast injection. The automated scan function of QT Ultrasound allows the operator to perform a fully automated breast scan without user interaction once the one button scan function has been selected. Data are automatically acquired and stored for postprocessing. This acquisition protocol enables temporal comparisons of data sets. Due to the prone positioning of the breast, 3D reconstruction, and type of images, the QT Ultrasound studies can be compared in the same plane and orientation as those acquired with other imaging modalities such as Magnetic Resonance Imaging (MRI).

QT Ultrasound uses a new inverse-scattering technology to provide quantitative sound speed mapping of breast tissue. This speed mapping is converted into a 3D breast image, and the map data also serves as a data set to create a novel refraction-corrected compounded reflection image. The refraction correction for the reflection image is also carried out in 3D. Transmission imaging provides information about the type of breast tissue traversed, based on the speed of sound value in the specific voxel volume. Over the past 30 or more years, tomographic ultrasound inverse-scattering imaging methods of the breast have been developed; however these methods use two-dimensional or linearization techniques to solve what is inherently a nonlinear and three-dimensional problem. It is now clear that the range of tissue properties encountered in the breast is sufficiently large that linear approximations lead to artifacts, distortions, and inadequate spatial resolution. Until recently, the engineering technology and mathematical methods for full-wave inverse-scattering 3D tomography have been so complex that practical results in humans were not realized. Advances in algorithms and computing have made it possible to solve these complex equations and have resulted in the development of a clinically useful scanner [3–5]. QT Ultrasound Labs has developed a scanner for breast imaging that uses a multifrequency nonlinear 3D inverse-scattering algorithm. The QT Ultrasound system performs both reflection and transmission data acquisition, utilizing the transmission information to compute speed of sound maps which are diagnostically important on their own but which are also used to correct the reflection images for refraction effects. No other ultrasound system is able to perform these functions with the same accuracy and resolution, and this is the basis for the high performance of the system.

Digital XRM is the standard of care to screen women for breast health. There are recognized limitations in the definition and visualization of anatomic details with mammography and even further challenges of detecting lesions and accurate characterization of breast tissue in women with a dense breast pattern [6]. In order to provide better specificity in the diagnostic evaluation of breast lesions, QT Ultrasound has been developed to provide (1) a 3D technology to better visualize the anatomic components of the breast, (2) a standardized evaluation of the entire breast, and (3) biomarkers for improving the specificity of tissue and lesion characterization. Ultrasound imaging is particularly important in women with mammographically dense breast tissue, where the lack of contrast between dense breast tissue and breast abnormalities renders the mammogram less accurate [6]. In women with dense breast tissue, the ability to accurately characterize lesions is more difficult, leading to unnecessary biopsies and added patient anxiety. Traditional reflection B-mode ultrasound identifies more lesions than with screening mammography [7] but is also associated with more false positive results, leading to higher health care expenditures and more patient concern. Therefore, there is a need to streamline and standardize the ultrasound exam, provide more accurate anatomic information, and ultimately improve tissue characterization and increase lesion specificity.

QT Ultrasound, using transmission and reflection techniques, has been developed to address these shortcomings with conventional XRM. The QT Ultrasound technology has shown accurate spatial registration, high spatial and contrast resolution, and quantitative speed of sound measurements distinguishing different types of breast tissue. This study represents the first attempt to describe breast anatomy using high resolution 3-dimensional sonographic transmission imaging and correlate the images with cross-sectional anatomy of the same breast, validate speed of sound as an anatomy identifier, and compare QT Ultrasound and digital mammography in a visual grading study of 10 anatomic features of the breast.

## 2. Materials and Methods

### 2.1. Study Oversight

Cadaver breasts were purchased from The Life Legacy Foundation (Tucson AZ) which verified that all necessary donor consents were obtained. All breast cadaver specimens were returned to Life Legacy Foundation at the end of the study. The acquisition of normal volunteer breast images at QT Ultrasound Labs was under a protocol approved by the Western Institutional Review Board Research Foundation (Puyallup, WA). The mammograms and QT Ultrasound images for the VGA study were part of a case collection study in 2009 and 2010 approved by the three institutions involved (University of California, San Diego, The Orange County Breast Care and Imaging Center, Orange, CA, and the Mayo Clinic, Rochester, MN). The study was designed by all the authors. All the authors collected the data. The initial independent data analysis was conducted by the first, second, and third authors. Subsequent interpretation and analyses were conducted by all authors. All authors take responsibility for the accuracy and completeness of the analyses. The first author wrote the first draft of the manuscript; no one who is not an author contributed to the writing of the manuscript. All the authors made the decision to submit the manuscript for publication.

### 2.2. Cadaver Breasts

A total of 3 cadaver breast specimens provided the material for this part of the study. All cadaver specimens were obtained from a commercial provider (Life Legacy Foundation, Tucson, AZ) after appropriate donor consent. The specimens were embalmed in 10% buffered formalin and frozen at −20°C. Prior to sectioning all specimens underwent QT Ultrasound imaging as described below. Just prior to sectioning the specimens were deep frozen at −100°C in an alcohol-dry ice bath and sectioned using a butcher's band saw. Sections were cut at 1 cm intervals in the coronal plane starting at the nipple, and the section surfaces to be photographed were sanded with 300-grit sandpaper using a commercial orbital sander until a smooth, glistening surface was achieved and all the anatomic detail was optimally visualized. Sections were immediately photographed. Some of the structures such as vessels and nerves in the breast were not clearly visible because they were either collapsed or blended in with surrounding connective tissues. Biopsies were taken of all the major anatomic tissue types of the breast (skin, subcutaneous tissue, Cooper's suspensory ligaments, glandular tissue, regional ductal tissue and subareolar ductal tissue, and pectoralis muscle) and samples were sectioned and stained with hematoxylin and eosin prior to microscopy to confirm the microanatomy.

### 2.3. QT Ultrasound Imaging

The QT Ultrasound scanner uses a transmitter array emitting broad-band plane pulses (0.3–2 MHz) while the receiver array, comprising 1536 elements in 8 vertical rows, digitizes the time signal. A full data set consists of 50 or more (up to 70) overlapping levels of data, depending on breast size, which are acquired 2.0 mm apart. An algorithm is utilized that simulates wave propagation in 3D and inverts for a full 3D representation of sound speed and attenuation [5]. Unlike a 2D algorithm where one level of transmitted data is used, in a 3D inversion, multiple levels of 3D data are used to simultaneously invert multiple levels of the breast by the algorithm that propagates simulated 3D waves in a computational grid that extends above and below the data levels approximately 32 mm. The resulting image consists of voxels that are 0.65 mm × 0.65 mm in the horizontal and 1 mm in the vertical direction with a measured spatial resolution of 0.75 mm × 0.75 mm × 1.5 mm full width at half maximum (FWHM). The accuracy of the sound speed measurements by QT Ultrasound has been validated with phantoms of known composition. Highly reproducible sound speed accuracy and linearity can be found (*R* ^2^ = 0.992) over the range of boundary values of the algorithm. Sound speed contrast sensitivity is 3.5 m/sec. For the sound speed image, the full-width half maximum of the point spread function is 0.75 mm in plane with ~1.5 mm slice sensitivity profile at 1.25 MHz. The QT Ultrasound system also provides reflection tomography (RT) imaging. These images are refraction corrected and attenuation calibrated. RT data are collected with three horizontal reflection transducers that acquire data in 6° steps as they rotate 360°. The three RT transducers have three focal lengths which provide a large depth of focus when combined. The RT spatial resolution is 0.8 mm × 0.8 mm × 2.5 mm. Due to the high relative contrast, it is possible to resolve objects as small as ~300 *μ*.

### 2.4. Correlative Imaging

Photographic images of the coronal plane cut sections of the cadaver breast were correlated with the QT Ultrasound image coronal slices and eight gross anatomic features were identified on the cadaver cut sections: skin, Cooper's ligaments, and subcutaneous fat (with fat lobular structure detail visible); superficial veins, subareolar ducts, and TDLU (visible in the dense areas of the breast); intermediate ducts and pectoralis muscle. Cadaver sections were photographed with and without 10x magnification (using a dissecting microscope) to define smaller structures (down to 1 mm). Representative tissue samples were taken for histological analysis. Tissue biopsies were sent to a commercial histology laboratory (Borsting Labs, Novato, CA), stained with hematoxylin and eosin and mounted for visualization.

### 2.5. Speed of Sound Correlations

In order to verify the speed of sound for six histologically confirmed areas of the normal adult cadaver breast (i.e., fat, pectoralis muscle, ductal tissue, glandular tissue, skin, and connective tissue), 3 normal adult cadaver breasts fixed in 10% formalin were imaged and frozen at −20°C and processed as described above. Each breast was then sliced in the coronal plane into 1 cm thick slices and was photographed and selected areas of tissue were taken for histopathology. Areas of confirmed skin, fat, muscle, ductal, glandular, and connective tissue were then correlated with areas on the QT Ultrasound slices using microscopic analysis. One hundred measurements of speed of sound were taken and averaged for each tissue type. Mean and 1 SD of speed values on the cadaver breasts were calculated for each tissue type. In order to correlate cadaver and* in vivo* measurements, one hundred fifty similar measurements were taken on clinical QT Ultrasound images from 4 normal volunteers from IRB-approved case collection studies. Mean and 1 SD of speed values on the normal volunteers' breasts were calculated for each tissue type. Results of cadaver and normal volunteer breasts were pooled and plotted as scatter plots.

### 2.6. Tissue Classification Using Discriminant Analysis

While the transmission characteristics of the living breast tissue from normal volunteers show relatively specific range of speeds as a function of tissue type, as shown later below, further distinction between different tissue types can be made if the information from all the three modalities (speed of sound, reflection, and attenuation) provided by the QT Ultrasound scanner is used. Note that the respective images are perfectly coregistered in the spatial domain. Linear discriminant analysis (LDA) was used to classify the different normal breast tissue types [8]. Twenty-six data points were used for each normal breast tissue type: fat, ducts, glands, skin, and Cooper's ligaments. A leave-one-out cross-validation approach was adopted in order to assess the classification performance.

### 2.7. Image Quality Comparison Study of QT Ultrasound versus XRM in Living Subjects

The primary objective was to compare radiologists' (readers') subjective assessment of the image quality of ten normal breast structures as visualized on XRM versus QT Ultrasound. A relative image quality evaluation based on ordinal ratings was performed using visual grading analysis (VGA). XRM and QT Ultrasound images were taken from 22 normal breasts (a total of 44 image sets) from living subjects who were selected from an IRB-approved case collection study from three institutions (University of California San Diego, The Mayo Clinic, Rochester MN and The Orange County Breast Care and Imaging Center, Orange CA). All cases consisted of XRM and QT Ultrasound images for the same breast for which the XRM was available. Two digital XRM views (i.e., craniocaudal (CC) and mediolateral oblique (MLO)) were selected and the corresponding QT Ultrasound scans for the same subject including the entire DICOM study with transmission and reflection images in the coronal, axial, and sagittal planes were provided to the readers for evaluation. One blinded reading session was conducted. This was a paired-reader, paired-patient design comparing the image quality of QT Ultrasound to XRM. Four readers independently scored the image quality of 10 breast features (i.e., skin, epidermis, dermis, hypodermis, Cooper's ligaments, superficial veins, central ducts, intermediate ducts, TDLU, and pectoralis muscle) with XRM and QT Ultrasound independently. An overall image quality rating was also assigned. The following rating scale was used: 1 = excellent, 2 = good, 3 = sufficient, 4 = restricted, and 5 = poor.

All readers were Interpreting Physicians as defined under 21CFR 900.12(a)(1)(i)(B)(2); 3 met Mammography Quality Standards Act (MQSA) requirements and had annual review rate of at least 1,000 mammograms and 500 HHUS and all successfully completed a QT Ultrasound Reader Training program. One reader was an Interventional Radiology Fellow and completed a Breast Imaging Fellowship a year before. Standardized digital mammography workstations were used for all digital mammography interpretations during this multireader multicase (MRMC) study and all readers received training on the operation and the review functionality of the mammography workstation prior to performing study interpretations. A standardized digital workstation for review of QT Ultrasound images was used for all interpretations during this MRMC study and all readers received training on the operation and the review functionality of the workstation prior to performing study interpretations. All readers received training on review objectives, case review process, and scoring methodology. In addition, a tutorial was conducted for all readers on the use of the score rating scale. Readers were trained in the use of the Case Report Forms (CRF). A practice session with the paper CRF at the workstations was conducted with all readers prior to the initiation of the reading session to ensure understanding of the CRF.

The median image quality score for each of the 10 features, as well as the overall image quality, was reported for each reader. The proportion of breasts where the image quality was rated better on QT Ultrasound than XRM or equivalent to XRM was reported for each feature. A 95% Confidence Interval (CI) for the proportion of breasts rated as equivalent or better image quality on QT Ultrasound was constructed for each feature using methods for clustered binary data [9]. When the estimated proportion was one, the rule of three was used to construct the lower 95% confidence bound [10] using an approximation of 67 for the effective sample size (based on 22 breasts, scored by four readers, and using a conservative estimate of the intracluster correlation coefficient of 0.10). Similarly, the proportion of breasts where the image quality was rated better on QT Ultrasound than XRM was reported for each feature, along with its 95% CI.

## 3. Results

### 3.1. Sectional Anatomy with Correlated Imaging

**Figure 1:**
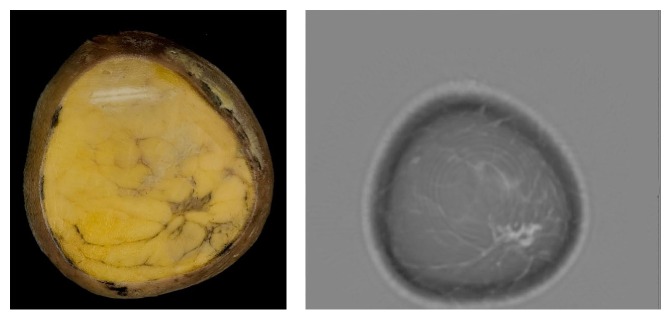
Cadaver cut surface photograph (left) and corresponding transmission (speed of sound) image (right) of the subareolar region. The tissue (dark on the left image and light on the right image) represents ducts, vascular structures, and some supporting connective tissue.

**Figure 2 fig2:**
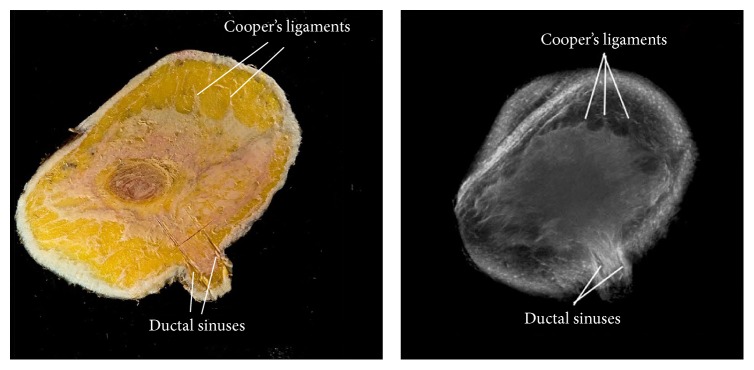
Cadaver cut surface photograph (left) and reflection image (right) at the areola. The tissue (white in both images) represents Cooper's supporting ligaments and fibroglandular tissue. The hypodermis (subcutaneous fat) is shown as black in the reflection image (right) and yellow in the cut section (left).

**Figure 3 fig3:**
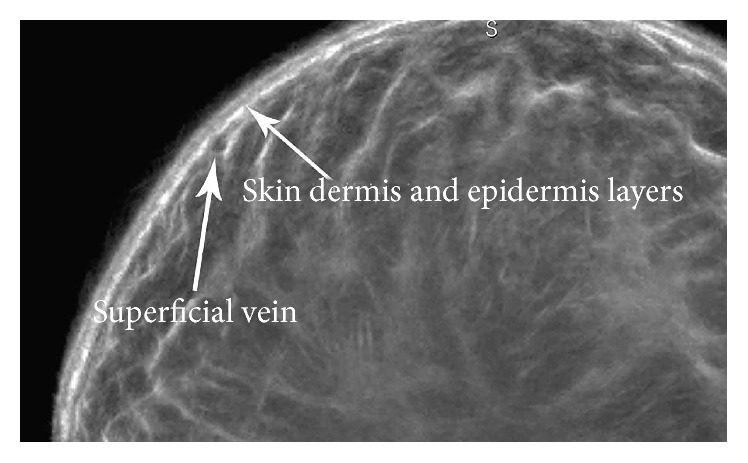
Magnified view of the reflection image at the skin surface showing the epidermal and dermal skin layers and a superficial vein in cross section.

**Figure 4 fig4:**
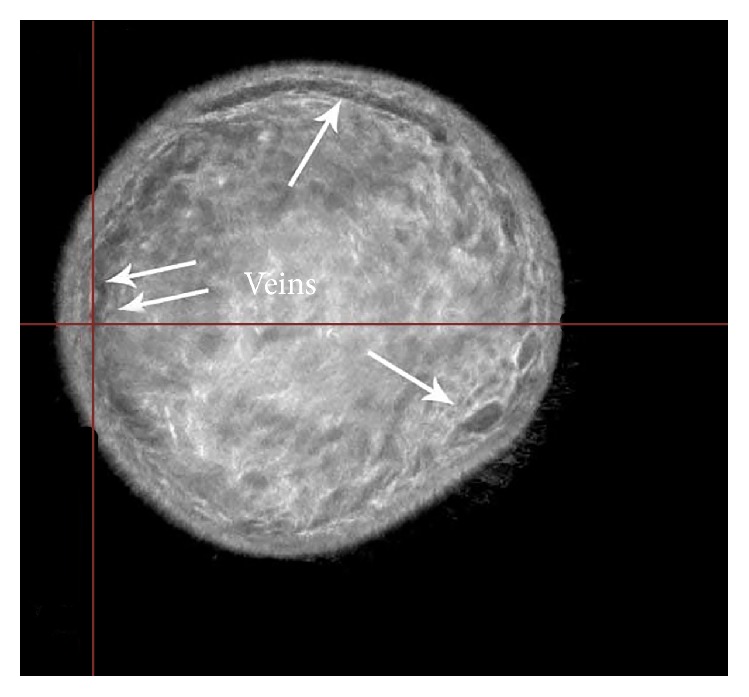
Reflection image of the breast showing superficial veins in cross section.

**Figure 5 fig5:**
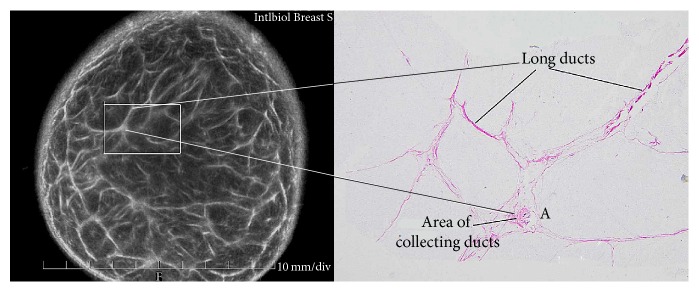
The tissue elements in the left reflection image (white) represent the supporting tissue of the breast ducts. These areas contain nerves, arteries, and ductal elements and are verified by histology (expanded image).

**Figure 6 fig6:**
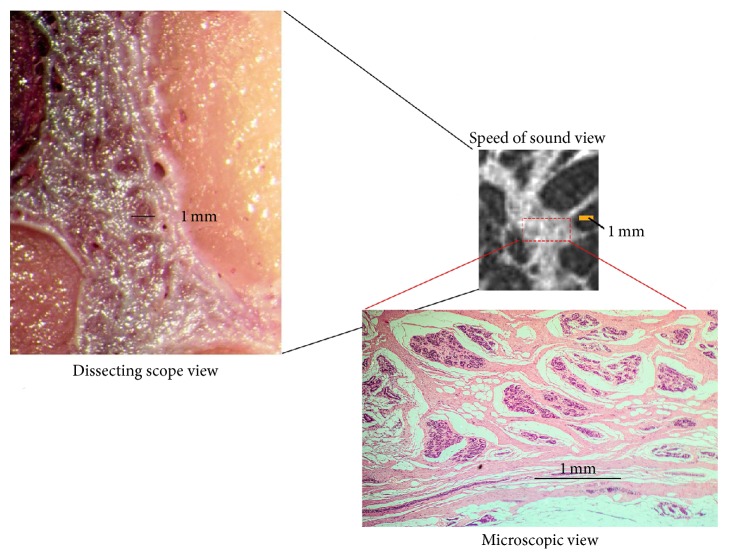
Higher magnification images of the cut surface of the cadaver breast (dissecting scope view) (left). The high magnification speed of sound image (upper right) of this same area and the results of biopsy of this same area showing glandular elements verified by histology (microscopic view). Solid bars represent 1 mm.

**Figure 7 fig7:**
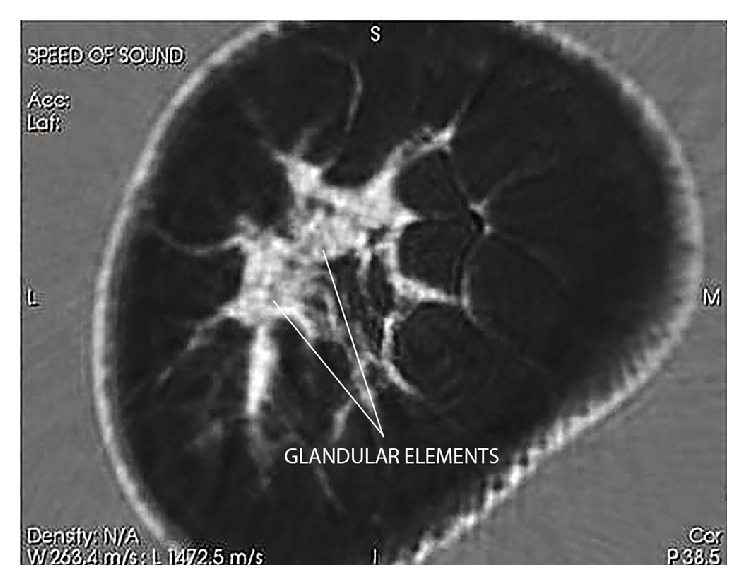
The transmission (speed of sound) coronal image is able to differentiate between glandular elements (speed values 1517–1567 m/s) from ductal tissue elements (speed values 1560–1612 m/s) in dense breast tissue as shown here in a moderately dense cadaver breast. The “stippled” appearance of the high magnification speed images differentiates the glands (grey) from the ductal and connective tissue elements.

**Figure 8:**
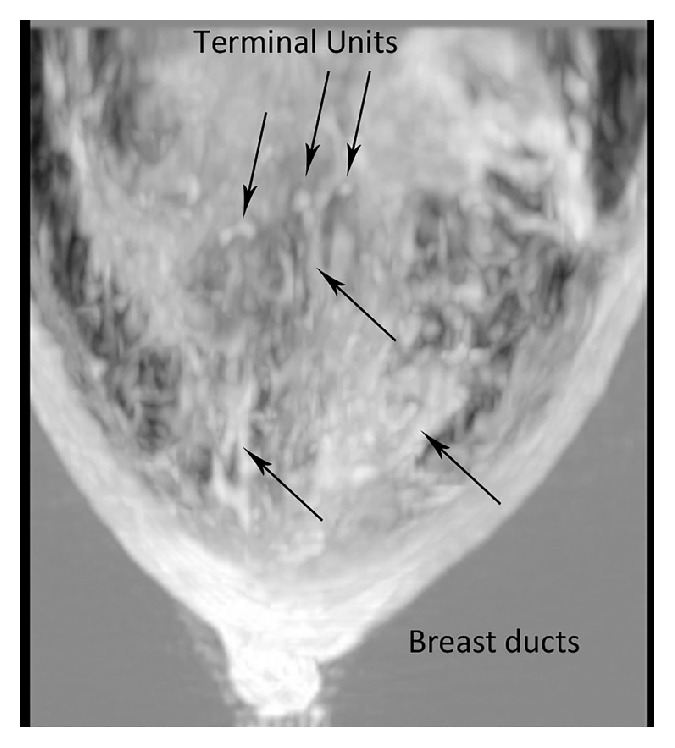
A craniocaudal (axial) view of the transmission (speed of sound) image as shown here in a woman with a dense breast. The relationship between the ducts and the TDLU can be better appreciated in this view.

The cut-section correlations between QT Ultrasound transmission and reflection images and the microscopic correlations are shown in Figures 1 [Fig fig2] [Fig fig3] [Fig fig4] [Fig fig5] [Fig fig6] [Fig fig7]–8. The connective tissue elements (white in both images) represent Cooper's supporting ligaments and the supporting tissue of the breast ducts. The hypodermis (subcutaneous fat) is shown as black in the reflection image (right) and yellow in the cut section on the left. A small artery is seen in cross section in the reflection image and verified by histology (expanded image).

### 3.2. Validation of Speed of Sound as a Tissue Identifier

**Figure 9:**
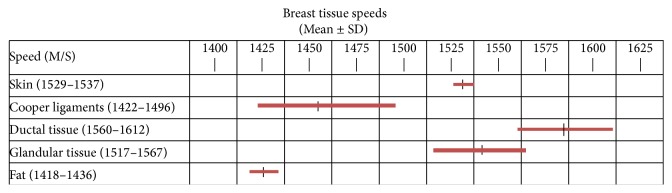
Summary analysis of 250 tissue measurements of speed of sound taken from 3 cadavers and four normal volunteers. Bars represent mean and 1 SD.

**Figure 10:**
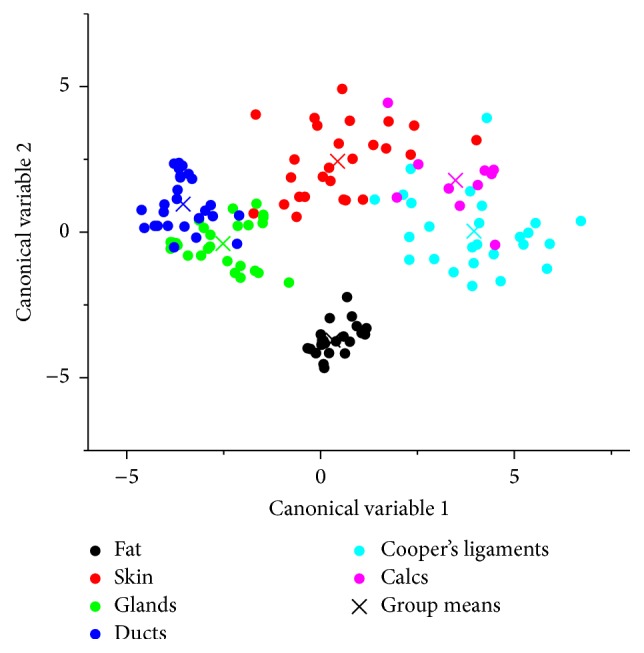
Results of the statistical linear discriminant analysis (LDA) classification based on the three QT Ultrasound features (speed of sound, attenuation, and reflection). The plot shows the projection of the data points onto the two-dimensional discriminant score space.

Figure 9 shows the summary analysis of 100 measurements of speed of sound taken from 3 normal cadaver breasts plus 150 similar measurements taken on four clinical QT Ultrasound images from normal volunteers. Differences between the cadaver values and the clinical subjects (not shown) were 4% for veins, 1.5% for fat, 1.5% for skin, 0.02% for glandular tissue, and 0.5% for ductal tissue. The speeds were slightly higher in cadaver fixed tissues. Results are combined in Figure 10.

### 3.3. Classification of Living Breast Tissue

As mentioned above, data from the three modalities was used to design a multiclass LDA classifier for the living subjects. The scatter plot shown in Figure 10 shows the projection of data points onto the 2D linear discriminant score space. Data belonging to different tissue classes are denoted by color. In general, there is weak overlap between classes indicating good performance of LDA.

The tissue classification is summarized in the confusion matrix shown in Table 1.

The overall accuracy in tissue classification was 88.5%. Note that while skin tissue can be classified well as a separate group, the skin tissue (and the surrounding water content) in the QT Ultrasound images can be segmented and eliminated from the images and therefore the classifier would not need to “detect” and “classify” skin tissue. If skin is excluded from the classifier, the overall accuracy is further improved to 91.4%.

### 3.4. Visual Grading Analysis

Table 2 summarizes the median image quality scores by reader and modality for each of the breast features from live subjects.

**Figure 11:**
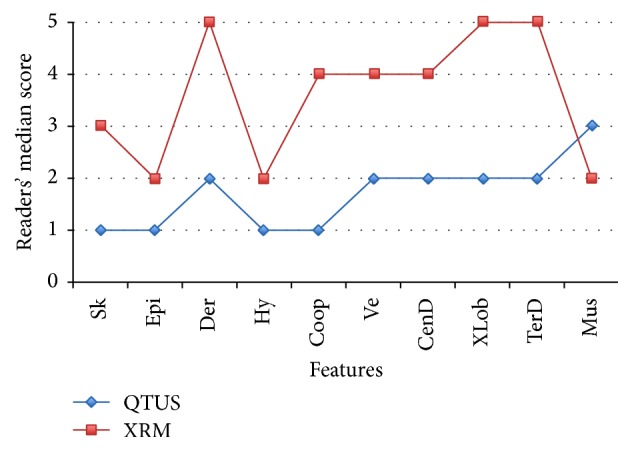
Readers' median image quality score by modality (QT Ultrasound in blue and X-Ray Mammography or XRM in red) and breast feature: skin, epidermis, dermis, hypodermis, Cooper's ligaments, superficial veins, central ducts, extralobular ducts, terminal duct lobular units, and pectoralis muscle. 1 = excellent; 5 = poor.

Figure 11 shows the median image quality scores by modality for each of the breast features as seen by QT Ultrasound (blue line) and X-ray mammography (red line). Except for muscle, the readers' median scores indicated superior image quality on QT Ultrasound.

Table 3 summarizes the proportion of breasts rated as equivalent or better image quality on QT Ultrasound than XRM for each feature, along with the 95% CI for the proportion. Except for pectoralis muscle, the readers scored the image quality on QT Ultrasound as equivalent or better than on XRM on each feature in more than 90% of breasts. Readers scored the image quality of pectoralis muscle on XRM as better than QT Ultrasound in almost 60% of breasts.

Table 4 shows the estimated proportion of breasts with better image quality on QT Ultrasound by feature. The readers scored the image quality on QT Ultrasound as better than on XRM in 69%–78% of breasts for epidermis, hypodermis, and skin, in 88%–90% of breasts for superficial veins and central ducts, and in ≥97% for dermis, Cooper's ligaments, extralobular ducts, terminal duct lobular units, and overall visualization of breast anatomy. The image quality of the muscle was rated superior on QT Ultrasound in 20% of breasts.

## 4. Discussion

This work presents the first effort to compare cross-sectional anatomy of the human female breast and QT Ultrasound images. We have validated QT Ultrasound as an accurate anatomic visualization method of the breast. We have also validated speed of sound as a tissue-specific identifier (biomarker) in breast images. All major anatomical features of the breast were identified by QT Ultrasound imaging analysis and validated using discriminate classifier analysis and visual grading analysis. QT Ultrasound can perform true 3-dimensional image acquisition and true 3-dimensional reconstruction of the breast at high resolution without ionizing radiation, compression, or contrast injection. The technique enables temporal comparisons of data sets and due to the prone positioning of the breast, the QT Ultrasound images can be compared in the same plane and orientation as those acquired with other imaging modalities such as MRI.

A significant problem in breast imaging is seeing anatomical detail in the dense breast. This occurs for mammography, hand-held ultrasound, and MRI. It is very difficult with any of the current methods to identify glandular detail in the dense breast (as defined by mammography). We believe that transmission ultrasound has made progress in being able to identify glandular detail in the dense breast. In future publications we plan to explore more detailed correlation with large-format and 3D histology as well as micro-CT and high-field MRI.

As Figure 7 shows, QT Ultrasound speed of sound images can show glandular detail. The ability to see distinctive features such as the ducts (bright areas) and in between these bright areas, the glandular elements (darker areas), is an advance in the ability to define detail within these heretofore hard-to-visualize parts of the breast. We believe that QT Ultrasound may be important in women with mammographically dense breast tissue, where the lack of contrast between dense breast tissue and breast abnormalities renders the mammogram less accurate. We are currently carrying out controlled clinical studies of sensitivity, specificity, call back rates, and biopsy rates in women with dense breasts to determine the clinical value of QT Ultrasound in the current breast imaging environment.

## Figures and Tables

**Table 1:** Classification matrix for the LDA of different normal breast tissue types. The overall accuracy of the separation of tissues using LDA was 88.5%.

		Fat	Skin	Glands	Ducts	Cooper's ligaments
Actual group	Fat	26	0	0	0	0
	Skin	0	24	1	0	1
	Glands	1	0	22	3	0
	Ducts	0	0	6	20	0
	Cooper's ligaments	0	3	0	0	23

**Table 2:** Median image quality scores by modality, reader, and feature.

	QTUS				XRM			
	R1	R2	R3	R4	R1	R2	R3	R4
Skin	1.5	1.0	1.0	2.0	2.0	4.0	3.0	2.0
Epidermis	1.0	1.0	1.0	1.0	2.0	4.0	2.0	1.0
Dermis	2.0	2.0	2.0	2.0	5.0	5.0	5.0	5.0
Hypodermis	2.0	1.0	1.0	1.0	3.0	4.0	2.0	1.0
Cooper's ligaments	1.5	1.0	1.0	1.0	4.0	4.0	4.0	4.0
Superficial veins	2.0	1.0	1.0	2.0	3.5	4.0	4.0	4.0
Central ducts	2.0	2.0	2.0	3.0	3.5	5.0	4.5	4.0
Extralobular ducts	2.0	2.0	1.0	2.0	5.0	5.0	5.0	5.0
TDLU	2.0	2.0	2.0	2.0	5.0	5.0	5.0	5.0
Pectoralis muscle	3.0	4.0	3.0	3.0	3.0	2.0	2.0	2.0
*Overall*	*2.0*	*2.0*	*1.0*	*2.0*	*4.0*	*4.0*	*4.0*	*4.0*

**Table 3:** Estimated proportion of breasts with equivalent or better image quality on QT Ultrasound by feature.

	Proportion of breasts with equivalent or better image quality on QT Ultrasound	95% CI
Skin	0.943 (83/88)	[0.898, 0.988]
Epidermis	0.920 (81/88)	[0.871, 0.970]
Dermis	1.0 (88/88)	[0.955, 1.0]
Hypodermis	0.943 (83/88)	[0.898, 0.988]
Cooper's ligament	1.0 (88/88)	[0.955, 1.0]
Superficial veins	0.989 (87/88)	[0.966, 1.0]
Central ducts	0.989 (87/88)	[0.966, 1.0]
Extralobular ducts	1.0 (88/88)	[0.955, 1.0]
TDLU	1.0 (88/88)	[0.955, 1.0]
Pectoralis muscle	0.409 (36/88)	[0.315, 0.503]
*Overall*	1.0 (88/88)	[0.955, 1.0]

**Table 4:** Estimated proportion of breasts with better image quality on QT ultrasound by feature.

	Proportion of breasts with better image quality on QT Ultrasound	95% CI
Skin	0.784 (69/88)	[0.710, 0.858]
Epidermis	0.693 (61/88)	[0.629, 0.757]
Dermis	0.989 (87/88)	[0.966, 1.0]
Hypodermis	0.705 (62/88)	[0.652, 0.757]
Cooper's ligaments	0.977 (86/88)	[0.947, 1.0]
Superficial veins	0.875 (77/88)	[0.805, 0.945]
Central ducts	0.898 (79/88)	[0.845, 0.950]
Extralobular ducts	1.0 (88/88)	[0.955, 1.0]
TDLU	0.989 (87/88)	[0.966, 1.0]
Pectoralis muscle	0.205 (18/88)	[0.115, 0.294]
*Overall*	0.966 (85/88)	[0.929, 1.0]
